# Effect of *Lactobacillus rhamnosus* HN001 on carriage of *Staphylococcus aureus*: results of the impact of probiotics for reducing infections in veterans (IMPROVE) study

**DOI:** 10.1186/s12879-018-3028-6

**Published:** 2018-03-14

**Authors:** Shoshannah Eggers, Anna K. Barker, Susan Valentine, Timothy Hess, Megan Duster, Nasia Safdar

**Affiliations:** 1 William S. Middleton Veterans Affairs Medical Center, 2500 Overlook Terrace, Madison, WI 53705 USA; 2 Department of Population Health Sciences, School of Medicine and Public Health, University of Wisconsin – Madison, Warf Office Bldg, 610 Walnut St #707, Madison, WI 53726 USA; 3 Department of Medicine, School of Medicine and Public Health, University of Wisconsin – Madison, 1685 Highland Ave, Madison, WI 53705 USA; 4 Division of Infectious Disease, Department of Medicine, School of Medicine and Public Health, University of Wisconsin – Madison, 1685 Highland Ave, 5th Floor, Madison, WI 53705 USA

**Keywords:** Clinical trial, MRSA, Lactobacilli, Probiotics, Veterans

## Abstract

**Background:**

Infection by *Staphylococcus aureus (S. aureus)* is a major cause of morbidity and mortality. Colonization by *S. aureus* increases the risk of infection. Little is known about decolonization strategies for *S. aureus* beyond antibiotics, however probiotics represent a promising alternative. A randomized controlled trial was conducted to determine the efficacy of *Lactobacillus rhamnosus* (*L. rhamnosus*) HN001 in reducing carriage of *S. aureus* at multiple body sites.

**Methods:**

One hundred thirteen subjects, positive for *S. aureus* carriage, were recruited from the William S. Middleton Memorial Medical Center, Madison, WI, USA, and randomized by initial site of colonization, either gastrointestinal (GI) or extra-GI, to 4-weeks of oral *L. rhamnosus* HN001 probiotic, or placebo. Nasal, oropharyngeal, and axillary/groin swabs were obtained, and serial blood and fecal samples were collected. Differences in prevalence of *S. aureus* carriage at the end of the 4-weeks of treatment were assessed.

**Results:**

The probiotic and placebo groups were similar in age, gender, and health history at baseline. *S. aureus* colonization within the stool samples of the extra-GI group was 15% lower in the probiotic than placebo group at the endpoint of the trial. Those in the probiotic group compared to the placebo group had 73% reduced odds (OR 0.27, 95% CI 0.07–0.98) of methicillin-susceptible *S. aureus* presence, and 83% reduced odds (OR 0.17, 95% CI 0.04–0.73) of any *S. aureus* presence in the stool sample at endpoint.

**Conclusion:**

Use of daily oral *L. rhamnosus* HN001 reduced odds of carriage of *S. aureus* in the GI tract, however it did not eradicate *S. aureus* from other body sites.

**Trial registration:**

ClinicalTrials.gov Identifier: NCT01321606. Registered March 21, 2011.

**Electronic supplementary material:**

The online version of this article (10.1186/s12879-018-3028-6) contains supplementary material, which is available to authorized users.

## Background

*Staphylococcus aureus* (*S. aureus*) is a well-known cause of infection and can lead to serious adverse health outcomes including mortality. Asymptomatic colonization by *S. aureus* greatly increases risk of infection [1–3]. The nares are the primary site of *S. aureus* colonization, however, *S. aureus* has also been found to colonize the gastrointestinal (GI) tract, oropharynx, and axillae of many individuals [4–6]. Those colonized by methicillin-resistant *S. aureus* (MRSA) are at even greater risk of subsequent infection than those colonized with methicillin-susceptible *S. aureus* (MSSA) [3, 7]. Thus reducing carriage of *S. aureus* is an important mitigating step in preventing infection.

Current treatment options for MRSA are limited to strong antibiotics that are accompanied by severe side effects and promote antibiotic resistance [8, 9]. Little is known about other *S. aureus* decolonization methods, however probiotics are emerging as a potentially low cost, well tolerated, and inexpensive alternative [10–14]. Probiotics are cultures of live bacteria species that normally reside in the human gut. They can help prevent infections by pathogens by outcompeting pathogenic bacteria for essential nutrients and mucosal binding sites, as well as enhancing immune function and promoting the production of mucosal and epithelial barriers [15]. *Lactobacillus rhamnosus* (*L. rhamnosus*) HN001, in particular has been shown to improve both innate and acquired immune function in multiple studies against a variety of bacterial infection threats [12, 13, 16–19]. It has been chosen for use in this study due to its immune effects and its safety and tolerability [20–22].

Given the paucity of data on the clinical use of probiotics to reduce *S. aureus* colonization [23], a randomized, double blind, phase II clinical trial was undertaken to evaluate the effect of probiotic use on *S. aureus* carriage. We hypothesized that the use of *L. rhamnosus* HN001, when compared to placebo, would decrease GI and extra-GI colonization by *S. aureus*, over a 4-week period.

## Methods

### Study design

The full protocol for this study has been previously published [24]. Study procedures and written consent form were approved by the University of Wisconsin Institutional Review Board and the Veterans Affairs Research and Development Committee. A total of 113 subjects were recruited from the William S. Middleton Veterans Affairs Medical Center in Madison, WI, and screened for *S. aureus* colonization at several body sites (Week 0). At the first study visit (Day 0, Week 1) subjects were stratified by site of colonization at baseline, either GI or extra-GI, and randomized into the probiotic or placebo groups. Those whose oropharyngeal, or perirectal swab tested positive for *S. aureus* via real-time polymerase chain reaction (PCR) assay only at baseline were included in the GI group. Those who tested negative for GI sites, and had at least one positive result from nasal, axillary, or wound swabs were included in the extra-GI group. Stratification assignment was based solely on PCR results, not results of the culture assays that were also performed. The first blood and stool samples were also collected at this visit. Contact with subjects was made weekly to collect adherence and adverse events data, as well a stool sample at the end of Weeks 1, 2, and 3. At the second study visit (Week 4), patients were re-screened for *S. aureus* using nasal, oropharyngeal, axillary, and wound swabs. Final stool and blood samples were also collected at Week 4. Presence of *S. aureus* and *L. rhamnosus* in stool was assessed at each of these time points. Participant compliance and adverse events were self-reported weekly throughout the study. Non-compliance was defined as missing at least one medication dose during the duration of the study.

### Enrollment criteria

Participants were eligible to enroll if they screened positive for *S. aureus* colonization, were at least 18 years of age, could take oral medication, and could consent to participation. A variety of exclusion criteria were used, including current MRSA decolonization or treatment regimens. A full list of inclusion and exclusion criteria are listed in the protocol [24].

### Intervention

The intervention consisted of once daily oral probiotic or placebo capsule administered for four weeks. The probiotic capsule contained 1 × 10^10^ colony forming units of *L. rhamnosus HN001* and an inert filler. Placebo capsules were identical in appearance and taste, but contained only the inert fuller. Probiotic and placebo capsules were provided by DuPont Nutrition and Health, Madison, WI.

### Microbiological analysis

Swabs and stool samples were analyzed for the presence of MSSA and MRSA using a real-time PCR assay, GeneXpert’s Xpert SA Nasal Complete kit (Cepheid, Sunnyvale CA), as well as traditional selective culture methods [25]. Cultures were grown using broth enrichment followed by standard microbiologic techniques including inoculation of selective media and identification of colony morphology [26]. Kirby Bauer Disk Diffusion testing was used to assess resistance to oxacillin [27, 28]. Stool samples were analyzed for the presence of *L. rhamnosus* HN001 using traditional culture methods followed by a strain specific in-house PCR.

### Statistical analysis

To assess demographics and adverse events, frequency tables with Fisher’s exact test were used to account for small cell sizes. To make results of this trial more generalizable to a broader population, and more useful for practitioners, data was analyzed by intention to treat. To assess the effect of 4 weeks of treatment on colonization by *S. aureus*, comparing probiotic and placebo groups, the Cochran-Mantel-Haenszel test for differences across strata was used, stratifying by baseline carriage site (GI or extra-GI). All data analysis was conducted in SAS version 9.4 (SAS Institute, Cary NC).

## Results

**Fig. 1:**
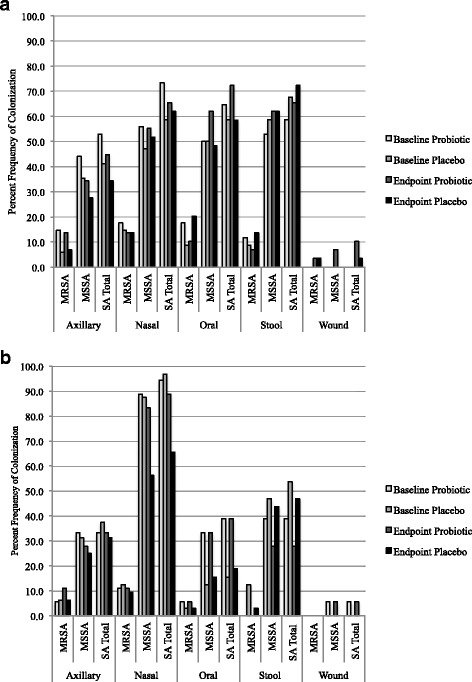
Percent frequency of methicillin-resistant *Staphylococcus aureus* (MRSA), methicillin susceptible *S. aureus* (MSSA), and total combined *S. aureus* (SA) colonization at endpoint and baseline of the probiotic and placebo groups within the gastrointestinal (GI) (**a**) and extra-GI (**b**) strata. Results shown are from both polymerase chain reaction (PCR) assays, and culture assays. **a** *Staphylococcus aureus* Colonization at Baseline and Endpoint in GI Group. **b ***Staphylococcus aureus* Colonization at Baseline and Endpoint in Extra-GI Group

The study population was predominantly male, with an overall average age of 63.6 years old (Table 1). The study groups had similar medical histories, although the placebo group had a higher frequency of current liver disease than the probiotic group (Table 1). At baseline, 80% of patients were colonized at more than one sampling site. The large frequency of participants in the GI randomization group who were colonized at axillary/groin, nasal, and wound sites show that many participants were colonized at both GI and extra-GI sites (Fig. 1).

**Table 1:** Distribution of demographics and medical conditions of study participants, stratified by treatment group

	Probiotic (*N* = 52) n (%)	Placebo (*N* = 61) n (%)	*P*-value
Age (mean), (Q1, Q2)	64.4 (55.5, 69)	62.9 (57, 68)	0.7322
Gender (Male)	49 (94.2)	56 (91.8)	0.7239
Ambulatory Status (Inpatient)	4 (7.7)	3 (4.9)	0.5633
Regular Probiotic Use^a^	17 (32.7)	22 (36.1)	0.8429
History of *C. difficile* Infection	1 (1.9)	1 (1.6)	1.0000
History of Gram Negative Bacilli Infection	5 (9.6)	9 (14.8)	0.5686
History of MRSA Infection	15 (28.9)	12 (19.7)	0.2763
History of MSSA Infection	3 (5.8)	5 (8.2)	0.7239
History of VRE Infection	0 (0.0)	0 (0.0)	1.0000
History of Candida Infection	3 (5.8)	1 (1.6)	0.3325
Ongoing Clinical Infection of Any Type	1 (1.9)	2 (3.3)	1.0000
Human Immunodeficiency Virus Infection	1 (1.9)	1 (1.6)	1.0000
Current Active Surgical Wound	4 (7.7)	2 (3.3)	0.4113
Current Active Cancer	2 (3.9)	2 (3.3)	0.5780
Current Coronary Artery Disease	23 (44.2)	22 (36.1)	0.5018
Current Diabetes	17 (32.7)	22 (36.1)	0.8429
Current Insulin Requirement for Diabetes	7 (13.5)	8 (13.1)	1.0000
Current Gastrointestinal Disease	27 (51.9)	30 (49.2)	0.9202
Current Hemodialysis	0 (0.0)	1 (1.6)	1.0000
Current Liver Disease	3 (5.8)	13 (21.3)	0.0282
Current Lung Disease	18 (34.6)	16 (26.2)	0.4114
Current Neutropenia	0 (0.0)	1 (1.6)	1.0000
Current Open Wound	2 (3.9)	2 (3.3)	0.6650
Current Peripheral Vascular Disease	7 (13.5)	8 (13.1)	1.0000
Current Renal Failure	2 (3.9)	3 (4.9)	1.0000
Current Skin Condition	14 (26.9)	11 (18.0)	0.2672
Current Alcohol or Drug Abuse	3 (5.8)	1 (1.6)	0.3325
Current Smoker	10 (19.2)	14 (23.0)	0.7040

*Abbreviations*: *Q* quartile, *C.* clostridium, *MRSA* methicillin-resistant *Staphylococcus aureus*, *MSSA* methicillin susceptible *Staphylococcus aureus*, *VRE* vancomycin resistant Enterococci

^a^This category includes all types of probiotics, and yogurts that may or may not include probiotics

One participant was lost to follow-up and none withdrew from the study (Table 2). Rates of non-compliance were similar among the study groups, although they were slightly higher in the placebo group. Although the frequency of missing a single treatment dose was relatively high in both groups, only one participant missed more than 25% of study doses. The self-reported use of probiotic products other than the trial drug was similar between treatment groups. Detection by PCR found that no subject in any treatment arm was colonized at baseline with *L. rhamnosus* HN001. At week 4, 51.9% of the probiotic group, and 1.6% of the placebo group were colonized with *L. rhamnosus* HN001. There were no significant differences in adverse events between the probiotic and placebo groups (Table 2). Overall, bowel function was the most common adverse event experienced in both treatment groups. Those in the GI group experienced more muscle cramping than the extra-GI group.

**Table 2:** Frequencies of study compliance and adverse events stratified by baseline colonization site and randomized treatment group

Compliance	Extra-GI (*N* = 50)			GI (*N* = 63)		
	Probiotic (*N* = 18) n (%)	Placebo (*N* = 32) n (%)	P-value	Probiotic (*N* = 34) n (%)	Placebo (*N* = 29) n (%)	*P*-value
Withdrawal/Lost to Follow-Up	0 (0.0)	1 (3.1)	1.0000	0 (0.0)	0 (0.0)	1.0000
Non-Compliant	3 (16.7)	7 (21.9)	0.7189	9 (26.5)	9 (31.0)	0.6699
Other Probiotic Use	7 (38.9)	11 (34.4)	0.5788	11 (32.4)	10 (34.5)	0.8418
Adverse Events						
Fever	0 (0.0)	2 (6.3)	0.5105	1 (2.9)	1 (3.5)	1.0000
Infection	0 (0.0)	1 (3.1)	1.0000	1 (2.9)	0 (0.0)	1.0000
Nausea/Vomiting	3 (16.7)	5 (15.6)	1.0000	4 (11.8)	2 (6.9)	0.6782
Constipation	4 (22.2)	2 (6.3)	0.1710	0 (0.0)	1 (3.5)	0.4603
Cough/Cold/Congestion	1 (5.6)	5 (15.6)	0.3991	3 (8.8)	6 (20.7)	0.2804
Headache	0 (0.0)	1 (3.1)	1.0000	1 (2.9)	1 (3.5)	1.0000
Muscle Pain/Cramp/Spasm	3 (16.7)	5 (15.6)	1.0000	11 (32.4)	4 (13.8)	0.1371
Upset Stomach/Heartburn	1 (5.6)	1 (3.1)	1.0000	1 (2.9)	1 (3.5)	1.0000
Gas/Bloating	2 (11.1)	1 (3.1)	0.2914	1 (2.9)	1 (3.5)	1.0000
Insomnia	0 (0.0)	0 (0.0)	1.0000	1 (2.9)	1 (3.5)	1.0000
Unusual Stool (Loose/ Discolored/More Frequent)	2 (11.1)	8 (25.0)	0.2947	7 (20.6)	5 (17.2)	1.0000
Bad Taste	0 (0.0)	2 (6.3)	0.5298	0 (0.0)	1 (3.5)	0.4603
Cardiovascular Event	0 (0.0)	2 (6.3)	0.5298	0 (0.0)	0 (0.0)	1.0000
Itchiness	0 (0.0)	0 (0.0)	1.0000	0 (0.0)	2 (6.9)	0.2079
Swelling	1 (0.0)	1 (3.1)	1.0000	0 (0.0)	0 (0.0)	1.0000

*Abbreviations*: *GI* gastrointestinal

**Fig. 2:**
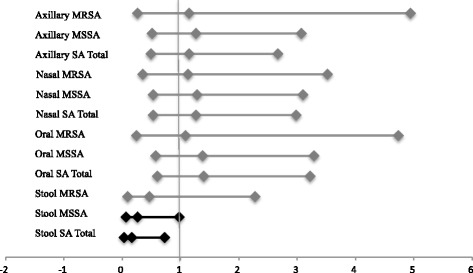
Cochran-Mantel-Haenszel odds ratios of methicillin-resistant *Staphylococcus aureus* (MRSA), methicillin susceptible *S. aureus* (MSSA), and total combined *S. aureus* (SA) colonization at different body sites, stratified by initial colonization site, either gastrointestinal (GI), or extra-GI, comparing the probiotic group to the placebo group

The prevalence of MRSA, MSSA, and any *S. aureus* in stool samples collected at the end of weeks 1, 2, and 3 were similar between the placebo and probiotic groups each week (data not shown). Stool sample colonization by MSSA and total *S. aureus* (combined MSSA and MRSA) prevalence of those initially only colonized at extra-GI sites was lower in the probiotic than placebo group by more than 15%, (Fig. 1). It is also notable that there was an approximately 30% reduction in nasal colonization by MSSA in the extra-GI placebo group between baseline and week 4 (Fig. 1). The odds ratio, adjusted for original colonization site, estimates that those in the probiotic group had 73% lower odds of MSSA presence, and 83% lower odds of any *S. aureus* presence in the stool sample at endpoint, as those in the placebo group (Fig. 2). No other sampling sites showed a significant difference in endpoint colonization between the probiotic and placebo groups (Fig. 2). When sampling sites were pooled and analyzed in categories of GI or extra-GI, there were no significant differences between probiotic and placebo groups (Table 3).

**Table 3:** Frequencies of MRSA, MSSA, and Total SA colonization at Week 4 by GI and extra-GI body site, stratified by site of original colonization, and randomized treatment group

Initial Colonization Site:	Extra-GI (*N* = 50)				GI (*N* = 63)			Cochran-Mantel-Haenszel	
Final Colonization Site	Organism	Probiotic (*N* = 18) n (%)	Placebo (*N* = 32) n (%)	P-value	Probiotic (*N* = 34) n (%)	Placebo (*N* = 29) n (%)	P-value	Odds Ratio	95% Confidence Interval
GI	MRSA	1 (5.6)	2 (6.3)	1.0000	5 (14.7)	4 (13.8)	1.0000	1.00	0.29–3.43
	MSSA	9 (50.0)	16 (50.0)	0.8368	23 (67.7)	21 (72.4)	0.9105	0.78	0.34–1.83
	SA Total	9 (50.0)	17 (53.1)	0.7588	25 (73.5)	24 (82.8)	0.7574	0.62	0.26–1.52
Extra-GI	MRSA	3 (16.7)	3 (9.4)	0.4485	5 (14.7)	4 (13.8)	1.0000	1.30	0.43–3.90
	MSSA	15 (83.3)	19 (59.4)	0.1332	16 (47.1)	16 (55.2)	0.8635	1.11	0.49–2.53
	SA Total	16 (88.9)	22 (68.8)	0.2728	20 (58.8)	19 (65.5)	0.6134	1.08	0.46–2.52

*Abbreviations*: *MRSA* methicillin-resistant *Staphylococcus aureus*, *MSSA* methicillin susceptible *Staphylococcus aureus*, *SA Staphylococcus aureus*, *GI* gastrointestinal

Because the original stratification of treatment was done using only PCR colonization results, some participants were stratified to the extra-GI group, but had positive cultures at GI sites. We undertook a sensitivity analysis in which we re-categorized these subjects by initial colonization site based on all swab and stool samples analyzed by both culture and PCR (instead of just by PCR). Twenty participants (10 placebo, 10 probiotic) originally included in the extra-GI randomization strata were moved to the GI strata in this analysis. While effect sizes of the odds ratios were similar to the original analysis, there was no statistical significance (Additional file 1).

## Discussion

*S. aureus* infections are a serious health risk with limited treatment options, and those who are colonized by *S. aureus* are at much higher risk of symptomatic infection. In this study, we observed that administration of *L. rhamnosus* HN001 over a four-week period reduced the odds of colonization in the stool sample, especially for those who were originally colonized outside of the GI tract. Probiotic treatment did not, however, reduce odds of colonization at other body sites. While the probiotic was not successful at eradicating *S. aureus* colonization at all body sites, it was associated with lower odds of colonization in the GI tract, especially for those who were stratified to the extra-GI group.

The surprising result of the reduction in nasal MSSA colonization in the extra-GI placebo group is likely explained by the natural history of *S. aureus* colonization. *S. aureus* is known to colonize intermittently and its presence is dependent on a number of factors including environmental exposures and other colonizing bacteria, to name a few [29–31]. Our study did not collect daily exposure data on participants to control for these external factors, which is why the placebo-controlled arm of the study was so important.

*L. rhamnosus* HN001 is thought to work in multiple ways. The primary potential mechanism is competitive inhibition, whereby colonizing the GI tract with healthy commensal microbes prevents the colonization of pathogens by out-competing them for vital resources [32]. This is likely the mechanism that led to differences in stool colonization between probiotic and placebo groups. A second potential mechanism is that *L. rhamnosus* HN001 has previously been shown to stimulate systemic immune function [12, 13, 18], making the host immune system more capable of eliminating *S. aureus* colonization at sites throughout the body. Based on the null results from body sites examined beyond the stool sample, it is likely that the *L. rhamnosus* HN001 used in this study population did not produce enough of a systemic immune response to affect *S. aureus* colonization at sites outside the gut. Further testing of phagocytic activity in the blood samples is currently being conducted to evaluate this hypothesis.

Our results differ from those of a study conducted in 2003 by Glück and Gebbers [14], which found that ingestion of *L. rhamnosus* GG, significantly reduced the occurrence of nasal carriage of potentially pathogenic bacteria. However, this discrepancy is likely due to the different strain of probiotic used in the intervention, a different delivery matrix used (yogurt/fermented milk product versus capsule), or inclusion of different *Staphylococcus* species in the outcome. There is a paucity of data on in vivo use of probiotics as a decolonization strategy for *S. aureus*, and more research is needed in this area.

This trial contributes critical knowledge to the literature about the efficacy of probiotics to reduce *S. aureus* colonization, however, it does include some limitations. Participants of this trial were drawn from the Veterans Affairs hospital system in Madison, WI. This system predominantly serves older men, thus the majority of the study sample were older men. Therefore, this sample may not be broadly generalizable to other populations.

The seven inpatients in this study were at increased risk of re-exposure to *S. aureus* during their hospital stay. It is possible these patients were cleared of their original *S. aureus* carriage, and re-colonized during the course of the trial. While stool samples were taken during the course of the intervention, no swabs of sample sites were taken between the baseline and endpoints of the trial. Thus, we cannot determine if any subjects were de-colonized and subsequently re-colonized at any of the swabbed sites between sampling. If this were the case for any of our sample, it would bias our results toward the null. The number of inpatients in each treatment group were not statistically different, therefore adjustment for ambulatory status in the analysis was not warranted. In the future, strain typing of stored isolates from stool samples can reduce this possibility of bias.

Considering that the only significant difference in health history between the treatment groups at baseline was prevalence of liver disease, we do not believe underlying health conditions affect the interpretation of trial results. Likewise, although overall non-compliance was around 30% in both treatment groups, all but one participant missed less than a quarter of the trial doses. Non-compliance with the assigned treatment would bias our results toward the null, and given the low number of doses missed by the majority of non-compliant participants, we do not believe this strongly biases the study findings. Use of other probiotics during the study may be more troublesome. Use of all possible probiotic supplements, including yogurts, capsules, etc., were considered as other probiotic use, however, we did not verify that reported probiotic supplements actually contained probiotics. The reported use of outside probiotics by approximately 1/3 of the placebo group could bias these findings toward the null by making the treatment groups more similar than otherwise expected. Future studies should carefully monitor the use of probiotics outside of study treatment.

## Conclusion

The results of our study support the use of this probiotic strain for gut decolonization by *S. aureus*, including MRSA. Further studies are needed to replicate the findings of this study and compare probiotics with other available agents used for decolonization such as antibiotics. Other directions for future research include examining the effect of alternative probiotic bacterial strains on *S. aureus* nasal colonization.

## Additional file

Additional file 1: ST1. Sensitivity analysis results: frequencies of MRSA, MSSA, and Total SA colonization at the endpoint of the trial at each body site, stratified by re-assigned GI or extra-GI colonization group based on PCR and culture screening results at baseline, and probiotic or placebo treatment group. (DOCX 110 kb)

## Abbreviations

GI: Gastrointestinal
*L. rhamnosus*: *Lactobacillus rhamnosus*
MRSA: Methicillin-resistant *S. aureus*
MSSA: Methicillin-susceptible *S. aureus*
PCR: Polymerase chain reaction
*S. aureus*: *Staphylococcus aureus*
