# Supplementary material for: Effect of Lactobacillus rhamnosus HN001 on carriage of Staphylococcus aureus: results of the impact of probiotics for reducing infections in veterans (IMPROVE) study
Source: BMC Infect Dis. 2018 Mar 14;18:129. doi: 10.1186/s12879-018-3028-6 (PMC5853063; doi:10.1186/s12879-018-3028-6)
Supplement: Supplementary file 1 — ST1. Sensitivity analysis results: frequencies of MRSA, MSSA, and Total SA colonization at the endpoint of the trial at each body site, stratified by re-assigned GI or extra-GI colonization group based on PCR and culture screening results at baseline, and probiotic or placebo treatment group. (DOCX 110 kb) [file 12879_2018_3028_MOESM1_ESM.docx]

| **ST1.** Sensitivity analysis results: frequencies of MRSA, MSSA, and Total SA colonization at the endpoint of the trial at each body site, stratified by re-assigned GI or extra-GI colonization group based on PCR and culture screening results at baseline, and probiotic or placebo treatment group. | | | | | | | | | | | |
| --- | --- | --- | --- | --- | --- | --- | --- | --- | --- | --- | --- |
| Initial Colonization Site: | | Extra-GI (N=20) | | | GI (N=93) | | | Cochran-Mantel-Haenszel | | | |
| Final Coloniza-tion Site | Organism | Probiotic (N=8), n (%) | Placebo (N=12), n (%) | P-value | Probiotic (N=44), n (%) | Placebo (N=49), n (%) | P-value | Odds Ratio | 95% Confidence interval | | |
| Axillary | MRSA | 1 (12.5%) | 0 (0.0%) | 0.1600 | 3 (6.8%) | 4 (8.2%) | 1.0000 | 1.12 | 0.26 | - | 4.82 |
|  | MSSA | 2 (25.0%) | 2 (16.7%) | 0.3136 | 15 (34.1%) | 14 (28.6%) | 0.8091 | 1.33 | 0.54 | - | 3.26 |
|  | SA Total | 2 (25.0%) | 2 (16.7%) | 0.3136 | 18 (40.9%) | 18 (36.7%) | 0.9636 | 1.21 | 0.51 | - | 2.85 |
| Nasal | MRSA | 1 (12.5%) | 0 (0.0%) | 0.1600 | 6 (13.6%) | 7 (14.3%) | 1.0000 | 1.14 | 0.37 | - | 3.51 |
|  | MSSA | 6 (75.0%) | 6 (50.0%) | 0.1577 | 25 (56.8%) | 27 (55.1%) | 0.4691 | 1.11 | 0.48 | - | 2.58 |
|  | SA Total | 7 (87.5%) | 6 (50.0%) | 0.6421 | 29 (65.9%) | 33 (67.4%) | 0.4270 | 1.09 | 0.48 | - | 2.46 |
| Oral | MRSA | 0 (0.0%) | 0 (0.0%) | 1.0000 | 4 (9.1%) | 4 (8.2%) | 0.8439 | 1.21 | 0.28 | - | 5.25 |
|  | MSSA | 1 (12.5%) | 2 (16.7%) | 1.0000 | 22 (50.0%) | 17 (34.7%) | 0.3319 | 1.60 | 0.68 | - | 3.77 |
|  | SA Total | 1 (12.5%) | 2 (16.7%) | 1.0000 | 26 (59.1%) | 21 (42.7%) | 0.2716 | 1.65 | 0.72 | - | 3.79 |
| Stool | MRSA | 0 (0.0%) | 0 (0.0%) | 1.0000 | 3 (6.8%) | 5 (10.2%) | 0.9005 | 0.64 | 0.14 | - | 2.96 |
|  | MSSA | 2 (25.0%) | 0 (0.0%) | 0.0760 | 23 (52.3%) | 32 (65.3%) | 0.1305 | 0.37 | 0.09 | - | 1.47 |
|  | SA Total | 2 (25.0%) | 0 (0.0%) | 0.0760 | 26 (59.1%) | 36 (73.5%) | 0.0502 | 0.26 | 0.06 | - | 1.23 |
| Wound | MRSA | 0 (0.0%) | 0 (0.0%) | 1.0000 | 0 (0.0%) | 1 (2.0%) | 0.7251 | * | -- | - | -- |
|  | MSSA | 0 (0.0%) | 0 (0.0%) | 1.0000 | 1 (2.3%) | 0 (0.0%) | 0.4731 | * | -- | - | -- |
|  | SA Total | 0 (0.0%) | 0 (0.0%) | 1.0000 | 1 (2.3%) | 1 (2.0%) | 1.0000 | * | -- | - | -- |
| * There were not enough data to calculate an odds ratio for colonization at wound sites.  Abbreviations: MRSA – Methicillin-resistant *Staphylococcus aureus*; MSSA – Methicillin susceptible *Staphylococcus aureus*; SA – *Staphylococcus aureus*; GI – Gastrointestinal. | | | | | | | | | | | |
